# Assessment of the aerobic glass beads fixed biofilm reactor (GBs-FBR) for the treatment of simulated methylene blue wastewater

**DOI:** 10.1038/s41598-020-77670-2

**Published:** 2020-11-26

**Authors:** Naresh Yadav Donkadokula, Iffat Naz, Anand Kishore Kola, Devendra Saroj

**Affiliations:** 1 Department of Chemical Engineering, National Institute of Technology Warangal, Warangal, Telangana India; 2 Centre for Environmental Health and Engineering (CEHE), Department of Civil and Environmental Engineering, Faculty of Engineering and Physical Sciences, University of Surrey, Guildford, Surrey, GU2 7XH UK; 3 Department of Biology, Scientific Unit, Deanship of Educational Services, Qassim University, Buraidah, 51452 Kingdom of Saudi Arabia

**Keywords:** Microbiology, Environmental sciences

## Abstract

The present research is focused on the application of glass beads (GBs) in fixed biofilm reactor (FBR) for the treatment of simulated methylene blue (MB) wastewater for 9 weeks under aerobic conditions. The COD of MB wastewater showed a reduction of 86.48% from 2000 to 270.4 mg/L, and BOD was declined up to 97.7% from 1095.5 to 25.03 mg/L. A drastic increase in the pH was observed until the 3rd week (8.5 to 8.28), and later, marginal changes between 8.30 ± 0.02 were noticed. A dramatic fluctuation was observed in ammonia concentration which increased (74.25 mg/L) up till the 2nd week, and from the 3rd week it started declining. In the 9th week, the ammonia concentration dropped to 16.5 mg/L. The color intensity increased significantly up till the 2nd week (259,237.46 Pt/Co) of the experiment and started decreasing slowly thereafter. The SEM–EDX analysis has shown the maximum quantity of carbon content in the GBs without biofilm, and then in the GB samples of 1st, and 9th-week old aerobic biofilms. Furthermore, Raman spectroscopy results revealed that the 9th-week GBs has a fine and strong MB peak and matched with that of the MB stock solution. Overall, the results have shown that the GBs filter media were suitable for the development of active biofilm communities for the treatment of dye wastewater. Thus, GBs-FBR system can be used for wastewater treatment to solve the current problem of industrial pollution in many countries and to protect the aquatic environment from dye pollution caused by the textile industry.

## Introduction

Water is an invaluable wealth, necessary for the existence of humankind. Safe drinking water for all the people across the globe remains a major challenge. Out of all the available water, a part is utilized and polluted by the industries, factories, agricultural fertilizers/pesticides, mining, power generation, etc^1^. Systematic utilization of the existing water and proper recycling/reuse of the used water is the need of the hour^2^. Across the globe, half of the available water is being used by the industries, which needs to be treated well so that it can be reused. Moreover, during the process of product development few industries such as pharmaceutical, textile, metal plating, chemical, etc. are releasing hazardous substances into the environment along with the effluent and eventually pollute the environment and cause several problems^3^.

The abundant quantity of chemicals and water are used in the textile industries in the product manufacturing^4^. Different dyes used by textile industries have different chemical structures; few dyes have complex chemical structures that can withstand the degradation in conventional treatment plants. Degradation of these complex chemicals is hard, expensive, and also time-consuming. Considering the environmental issues, uncompromising laws have been brought in many countries for dye removal from industrial effluents^5^. Several harmful pollutants exist in dye wastewater and some of them include acids, color, surfactants, urea, waxes, etc. Textile dyes are considered as visible pollutants, and they need to be treated well before its release into the environment^6^. Among various textile dyes, Methylene Blue (MB), a basic cationic dye, has predominant uses in many industries such as paper, dyeing, hair colorant, etc^7^. It is a threat to aquatic as well as terrestrial environments and has serious health consequences like quadriplegia, jaundice, cyanosis, diarrhea, increased heart rate, etc^8^. As the MB dye is recalcitrant, it can't be degraded easily in the environment^9^. Various methods involving physical, chemical, and biological methods are employed to remediate MB dye^10–13^.

Recent studies have revealed that the microorganisms are profitable alternatives, easily obtainable, environmentally friendly, sustainable, and cost-effective for dye degradation^14^. Hence the microbial consortium (biofilm) attached to a solid support medium was used as an efficient alternative method for the MB dye degradation. Many dyes having higher efficiencies can be decolorized by algae, fungi, and bacteria^15–17^. Out of these, under a wide range of external circumstances, bacteria are capable of gathering the contaminants, develop faster, and remain uncomplicated to culture compared with fungi and algae^18^. Among various biological technologies, biofilm-based mechanisms have overcome the drawbacks encountered by the regularly activated sludge systems^19^. The biofilm formation and its performance in treating the wastewater depend on the type of microbial communities and their activity^20,21^. Biofilm is the combination of a large number of microbial cell populations, which form a complex Extracellular polymeric substance (EPS) matrix. This complex matrix can be developed on a surface or even without any surface^22^. Solid support media play a crucial role in biofilm formation and act as a strong base for microbial attachments^20,23^. Various studies have reported several different types of support media for biofilm development such as pebbles, rubber, gravel, polystyrene, etc^24–26^. Further, the support media for biofilm configuration should be easily available, cost-effective, should not be easily blocked, permeable, and should have a large surface area^27,28^. Characterization of the support media and the biofilm development on it can be performed by some of the spectroscopic and microscopic methods. This includes X-ray photoelectron spectroscopy (XPS), Fourier transforms infrared (FTIR), Raman spectroscopy (RS), Scanning electron microscopy (SEM) and Energy-dispersive X-ray spectroscopy (EDX)^25,26,29^. Compared to the other support media, GBs are rigid and resistant to microbial communities developed as biofilms on it and also to the various contaminants in the wastewater^30,31^. Furthermore, various degradative enzymes produced by the microbial consortium act on the contaminants but not on the surface of the GBs. Hence, higher contaminant removal is possible using biofilms developed on GBs filter media^30,32^. Various industries are disposing a huge quantity of ineffectual glass and its byproducts into the environment frequently, and a proper disposal of glass waste is an expensive procedure^33^. These remnants can be used as a good resource for the biofilm development and its application for treatment of wastewater.

This research work is novel in terms of the study of a new aerobic bioreactor system, in which the GBs were used as the solid support media for the biofilm growth to treat the simulated MB wastewater. The performance of the system was evaluated through a detailed examination of physicochemical parameters, few microscopic, and spectroscopic methods during the experiment.

## Materials and methods

### Selection and characterization of the chemical composition of filter media

The GBs were selected as filter media for the development of biofilm and were washed thoroughly with the laboratory detergent. The surface area (4πr^2^) was measured with Vernier caliper and was found as 5.92 cm^2^. The elemental composition of the GBs was determined by X-ray photoelectron spectroscopy (XPS). Theta probe spectrometer (Thermo Fisher Scientific, East Grinstead, UK) was used for the analysis, and X-ray Al Kα monochromatic source (hν = 1486.6 eV, radius ~ 400 µm) was used to acquire the XPS spectra. The available sole elements in the GB media were obtained by passing 300 eV energy initially and 50 eV energy later^24,34^. After XPS characterization, the GB media were sterilized by autoclaving and transferred into the reactors for the configuration of biofilms.

### Experimental setup

**Figure 1:**
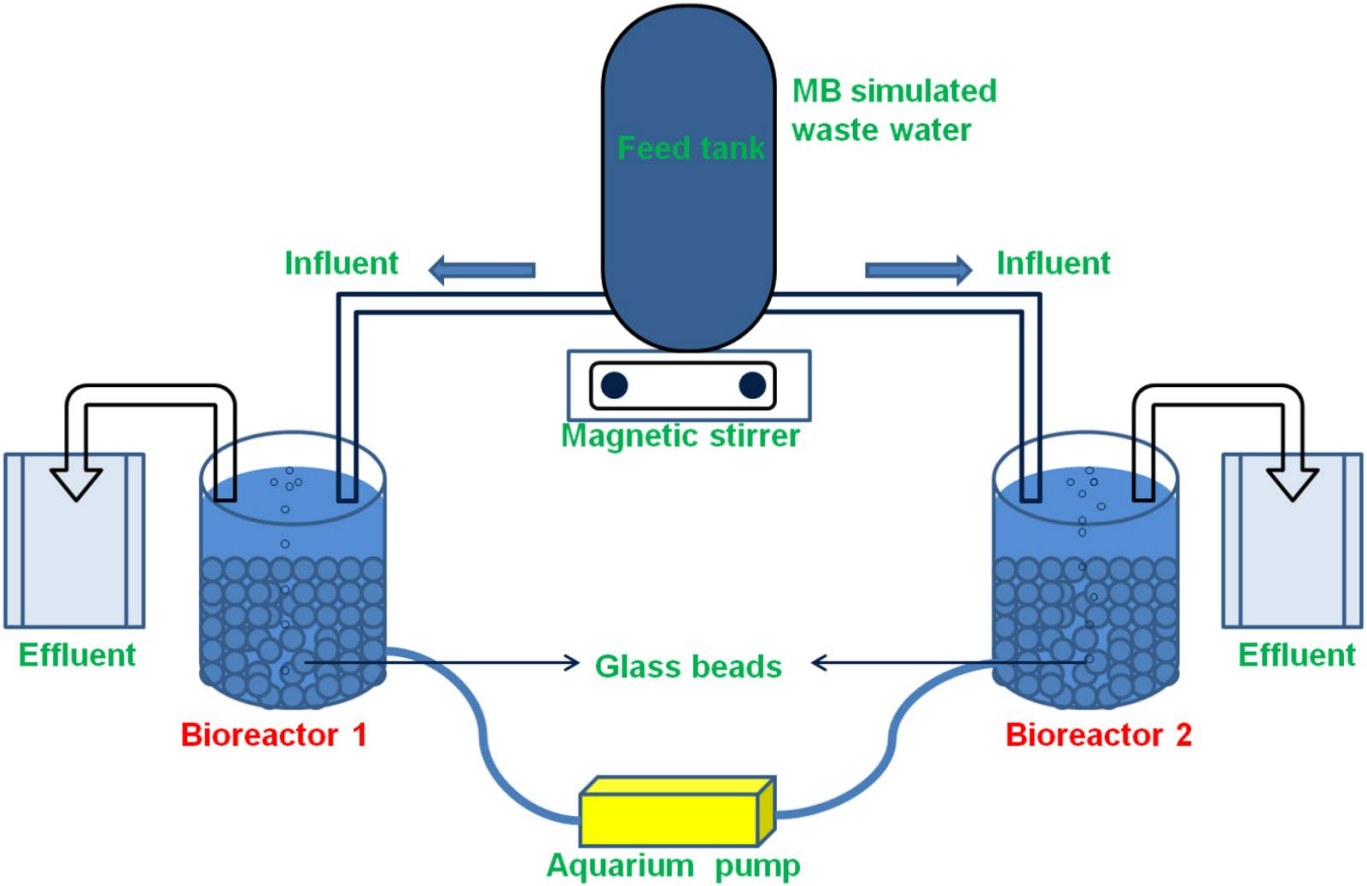
Schematic illustration of the experimental setup for simulated Methylene Blue (MB) dye wastewater treatment.

The schematic representation of the MB dye simulated wastewater treatment by aerobic fixed biofilm GBs reactor was shown in Fig. 1. Two glass jars each with a volume of 1000 mL were used as bioreactors (Table 1). This experiment was performed in triplicates in batch reactors in a fume cupboard under aseptic conditions for 9 weeks at room temperature. For maintaining aerobic conditions, Interpet aquarium air pumps (5 L/min)—AP4 provided with the stone diffusers were linked to the reactors (Fig. 1). After the evaluation of the chemical composition and sterilization, GBs were transferred into the reactors for the development of metabolically active biofilms on its surfaces. Clean Nalgene bottles of 1000 mL each were used for the collection of activated sludge samples from Guildford’s wastewater treatment plant (Surrey, UK), for the aerobic biofilm formation on the GBs surface. After the formation of physiologically active aerobic biofilms on GBs, 50 mL of simulated wastewater (influent) was transferred into the reactor and 50 mL of samples (effluent) were collected from the reactor every 2 days to maintain the consistency of the bioreactor.

**Table 1:** Configuration of the aerobic glass bead (GBs) fixed biofilm reactor (FBR) for simulated Methylene Blue (MB) wastewater.

Reactor parameters	Pilot-scale
Reactor volume	1000 mL
Void volume	310 mL
Hydraulic retention time (HRT)	55.03 days
GB surface area	5.92 cm^2^
COD loading rate	2000 mg/L

#### Composition and preparation of simulated methylene blue (MB) wastewater

MB stock (5 L) was prepared and mixed thoroughly using a magnetic stirrer. Then acetic acid of 1 N was used to adjust the pH to 8.5. The various chemicals present in the simulated MB dye wastewater were listed in Table 2, and the proportion of each of the chemicals is according to the previous research investigations^35,36^. In the present research study, MB dye was procured from M/s Sisco Research Laboratories Pvt. Ltd. Maharashtra, India, and other high purity grade chemicals were purchased from Sigma-Aldrich (Gillingham, United Kingdom).

**Table 2:** Composition of stock solution of simulated Methylene Blue (MB) wastewater.

S. No	Composition	Quantity (g/L)
1	Sodium hypochlorite	0.0224
2	Sodium chloride	0.007
3	Ammonium nitrate	0.1761
4	Magnesium chloride hexahydrate	0.0034
5	Calcium chloride dihydrate	0.004
6	Dibasic potassium phosphate trihydrate	0.0367
7	Sodium benzoate	0.1071
8	Sodium acetate	0.2049
9	Ferric sulfate heptahydrate	0.0084
10	Yeast extract	0.0084
11	Soy oil	0.224
12	Urea	0.420
13	Peptone	0.112
14	Methylene blue (dye)	5

#### Experimental operation

Initially, 100 mL of sludge sample containing various microorganisms was mixed with 500 mL of tap water and was transferred as feed to the GBs reactors under aseptic conditions. To acclimatize the microbial population in the reactors, on days 3rd, 5th, 7th, and 9th the supplementary sources (MB simulated wastewater) were injected gradually into the bioreactors in the ascending ratio of 1:4, 2:3, 3:2 and 4:1 respectively. After attaining acclimatization (11th day), 50 mL of the exclusive stock solution was transferred into the bioreactors on every second day. The microbial community present in the sludge enhances its population by using the supplementary sources. Soon after the beginning of the experiment under aerobic conditions, these microbial communities get attached to the surface of the media i.e. GBs in the form of a layer. This layer itself is a biofilm that degrades the dye present in the simulated wastewater. At fixed time intervals (every second day) supplementary organic sources (simulated wastewater) were transferred into the bioreactors. All the liquid and solid samples at regular intervals of time were characterized. Further, the development of biofim on GBs and its effect on dye degradation were regularly monitored. From the reactor, 50 mL of sample and two to three GBs were collected and transferred into the narrow mouth bottles for further physicochemical and biofilm characterization.

#### Physico-chemical characterization of simulated methylene blue (MB) influents and effluents

The standard methods such as 5220 D and 5210 B were used to analyze the COD and BOD_5_ of both the influent and effluent samples. Hach Spectrophotometry method 8025, embraced from SM-2120 C was employed to measure the true color. A bench-top pH probe (Hanna: HI 2210) was utilized to measure the pH according to standard method^37^. FTIR (Agilent Cary 640 FTIR) was used to assess the changes in MB simulated wastewater at 500 to 4000 cm^−1^ in triplicate. Using KBr pressed-disk technique the samples were scanned between 500 and 4000 cm^−1^ in triplicates^38^.

### Characterization of the surface of the glass beads (GBs) filter media

#### Scanning electron microscopic (SEM) analysis of the glass beads (GBs) filter media

The surface characterization of the GBs filter media was carried out by using SEM–EDX (JEOL JSM-7100F). Before the biofilm characterization by SEM, the collected samples from the bioreactors were saturated in 0.2 mol/L phosphate buffer. After thorough washing, the samples were dried (KADA 85 U/SMD) for 2 min and smeared with gold. The GBs filter media without biofilm, and GB samples of 1st, and 9th-week old aerobic biofilms were observed at 1000 and 3000 × magnification, under SEM^25,39,40^.

#### Raman spectroscopic analysis of the glass beads (GBs) filter media

The chemical, structural, and biological data of the biofilms were investigated by Raman spectroscopic analysis. The GBs without biofilms (raw), the GBs collected at different weeks after the development of biofilms, and the MB stock solution was characterized by Raman spectroscopy (RENISHAW in Via confocal Raman microscope of Rutherford Appleton Laboratory [RAL], United Kingdom). All the samples were collected in triplicates and characterized at 600–2000 cm^−1^ spectral interval, 10 s integration time, 5X objective extended scan, 830 nm wavelength, 5 accumulations, and 50 PC power.

#### Attenuated total reflection (ATR) analysis of the glass beads (GBs) filter media

Attenuated total reflection (ATR) was employed to investigate the changes taking place on the surface of the GB filter media due to the configuration of microbial communities. In this experimental study, raw GBs without biofilm were compared with the GBs collected at different weeks. Before the ATR analysis, the developed biofilms were scraped in 0.9% saline from GBs surfaces and then cleansed with distilled water.

### Ethics declarations

This article does not contain any studies with human participants or animals performed by any of the authors.

## Results and discussion

Currently, the production and utilization of the dyes for various purposes have increased enormously^41^. It is a known fact that the utilization of synthetic dyes are harmful to terrestrial and aquatic environments and will have a severe impact on all forms of life^42^. To control this terrible situation and to protect all forms of life on this earth from synthetic dye pollution, the textile industries are needed to adopt certain practices^43^.

### Estimation of the chemical composition of GBs filter media by X-ray photoelectron spectroscopy (XPS)

**Figure 2:**
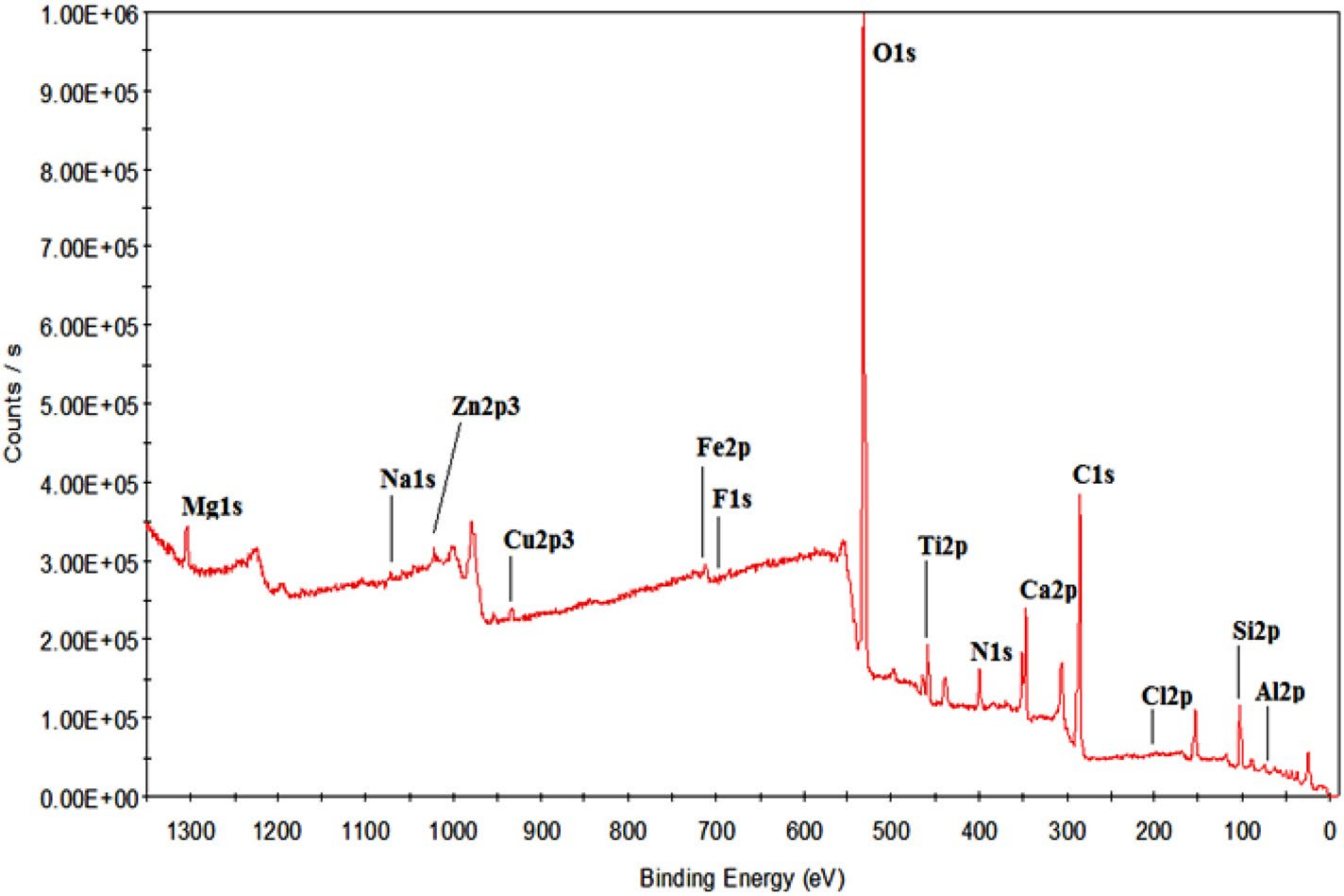
X-ray photoelectron spectroscopic (XPS) survey of glass bead (GB) surface before its employment in the fixed biofilm reactor (FBR) as filter medium.

The elemental composition of the GBs filter media was estimated using XPS. The elements identified over the surface of GBs are O (41.32%), C (35.86%), Si (7.83%), N (3.78%), Ca (3.51%), Al (2.38%), Ti (1.55%), Mg (1.54%), Fe (0.84%), Cu (0.38%), Na (0.36%), F (0.23%), Zn (0.22%) and Cl (0.20%). From the analysis, it was found that the GB media has huge amounts of oxygen, carbon, and also the nontoxic elements which are harmless for the biofilm development. Figure 2 explains the XPS survey spectrum of the GBs filter media surface samples. Several studies have revealed that the carbon, silica, and oxygen were identified as the major elements in GBs by XPS characterization^44,45^. Apart from this, the other peaks such as the Ti2p spectrum at 458.75 and 464.62 eV, Ti 2p1/2, and Ti 2p3/2 at 463.9 and 458.2 and a prominent peak at 530.13 eV indicating O1s spectrum were also observed^46,47^. With increasing the carbon concentrations, both the yield and growth rate were decreased in heterotrophic biofilm bacteria because of the carbon sources. Further, at low carbon concentration, the highest growth efficiency was observed^48^. Previously, it was found that the presence of oxygen, has a positive effect on the enhanced growth of the biofilm and it was also observed that the vicK mutant has remarkably enhanced the biofilm formation under aerobic conditions^49^. Few elements that are present in small amounts including silicon, zinc, nitrogen, etc. in the GBs can be used as biofilter media^25,50^. From this XPS analysis, it can be concluded that the GBs used in this research work has non-toxic elements and it can be recommended as a suitable solid support media for biofilm growth under aerobic conditions.

### Start-up phase of the glass beads (GBs) fixed biofilm reactor (FBR)

Enduring support media and metabolically active biofilms are crucial for effective wastewater treatment^51^. According to the previous literature, 3–60 days were reported to be required for biofilm development in a fixed-film bioreactor^28,52,53^. However, the start-up duration of the fixed-film reactor can be minimized by inoculating the activated sludge/commercial microbial cultures into the reactor^54,55^. The support media can be shifted into bioreactors after they were immersed in activated sludge for a certain period to reduce the start-up phase^34^. In the current experiment, the GBs were exposed to the activated sludge initially for the development of active biofilms, and then these developed biofilms act on the contaminants present in the simulated MB wastewater in the bioreactor. In another study, Yu et al.^28^ revealed that the start-up phase for wastewater treatment using grain slag media as 7 weeks. The utilization of the support media such as GBs, rubber, pebbles for the biofilm growth is one of the major reasons for the reduction in startup time, the microbial community gets quickly attached to the surface of the support media^56^. It was also reported in a study, that by using electrophilic suspended biofilm carriers, the reactor start-up time can be reduced^57^. In this study, the biofilm growth phases were classified into two types; primary (0–9 days) and secondary (9–13 days). While in 9 weeks of experimental work, several changes were observed in various phsicocemical parameters, due to microbial metabolic activity of biofims, as reported by another research group^58^, and the startup phase of the bioreactor was observed to be 11 days.

### Evaluation of the simulated MB wastewater treatment efficiency of the GBs-FBR by monitoring the physicochemical parameters of the influent and effluent

#### Chemical oxygen demand (COD)

**Figure 3:**
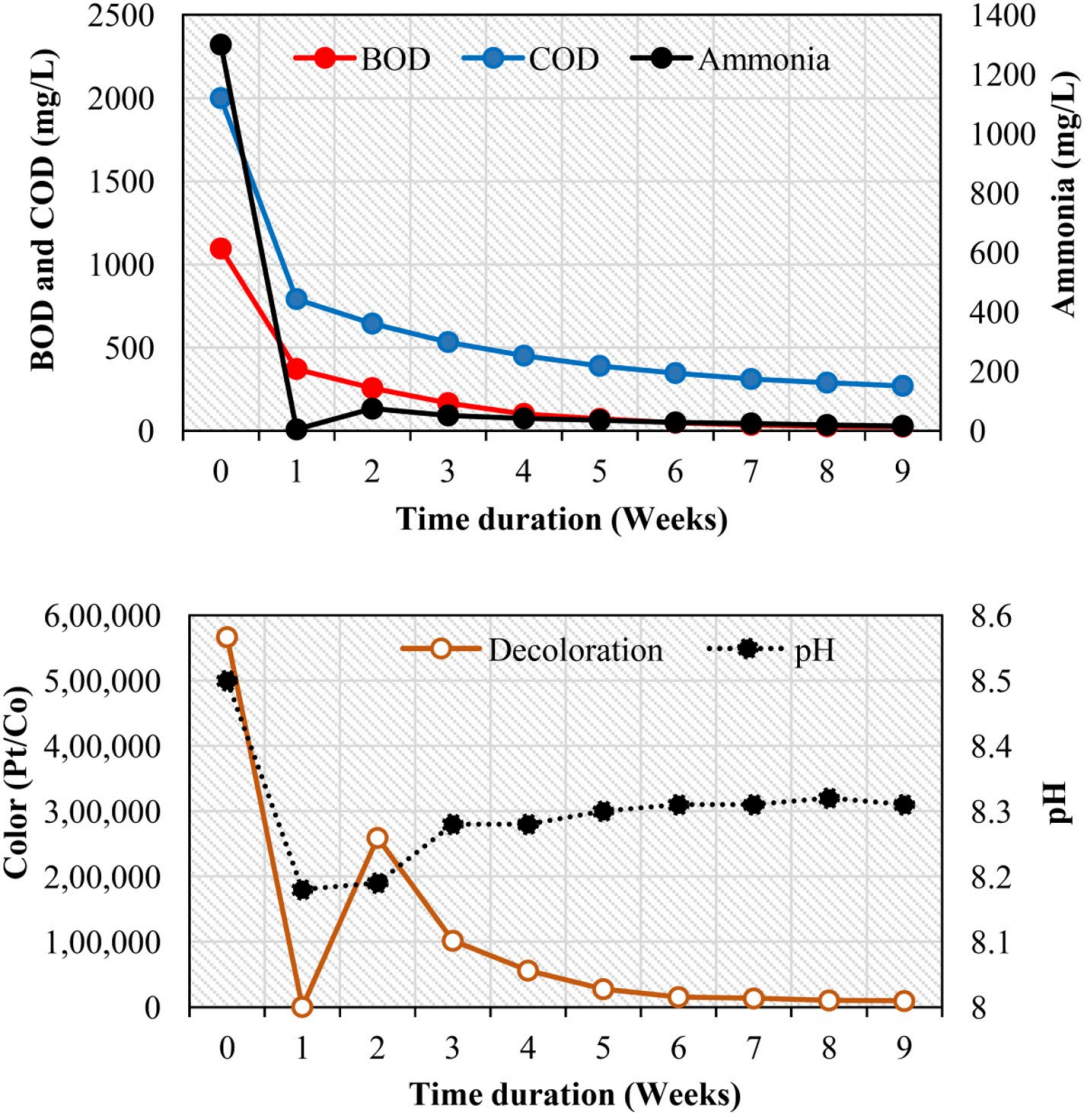
Changes in the levels of biochemical oxygen demand (BOD), chemical oxygen demand (COD), ammonia (NH_3_), pH and color intensity of the simulated Methylene Blue (MB) wastewater during nine weeks of treatment by GBs fixed bioreactor (FBR) under aerobic conditions.

The quantity of oxygen required to oxidize the organic carbon totally to CO_2_ and H_2_O is termed as COD. In this experimental work, COD was observed at fixed time intervals (weeks 0, 1, 2, 3, 4, 5, 6, 7, 8, and 9). The initial COD value of the sludge sample and simulated MB wastewater were found as 950 and 2000 mg/L respectively. The COD was reduced by 73.3% in 3 weeks and 86.48% in 9 weeks (last week). From the obtained results it can be concluded that a huge amount of COD reduction indicates a decrease in the organic pollutants due to active biomass in the reactor (Table 3 and Fig. 3). The higher biomass concentrations in the bioreactor resulted in highly significat COD (92.4%) removal of coke wastewater as reported previousely^59^. The biofilms can be developed on any support media including membranes^21,23^. Naresh et al.^26^ worked on MB dye removal using a biofilm TDR media reactor, under aerobic conditions and observed 89.2% of COD removal in 9 weeks. In another research study, under aerobic and anaerobic conditions, the biofilm developed on rubber media reduced the COD to 73.32 and 69.9% in 9 weeks^25^. Further, the biofilms were developed on the gas-permeable membrane for the treatment of synthetic wastewater, and it resulted in the removal of 95% of COD^60^. The higher COD reduction was due to the presence of a specific microbial population in the biofilm matrix present on the surface of filter media. A clear difference was observed in the COD reduction with and without the utilization of the support media for the development of biofim^61^.

**Table 3:** Physico-chemical characterization of the influent and effluent during 9 weeks of experiments in an aerobic glass beads (GBs) fixed biofilm reactor (FBR).

Physico chemical parameters	Sludge sample concentration	Influent characteristics	Effluent characteristics									Performance efficiency (% reduction)
			1st week	2nd week	3rd week	4th week	5th week	6th week	7th week	8th week	9th week	
COD (mg/L)	950	2000	792.0	644.5	533.5	451.7	390.4	347.0	311.16	289.5	270.4	86.48
BOD (mg/L)	62.2	1095.5	371.48	256.7	166.38	101.5	71.4	48.37	35.4	27.1	25.05	97.7
pH	6.2	8.5	8.18	8.19	8.28	8.28	8.30	8.31	8.31	8.32	8.31	**–**
Ammonia (mg/L)	20.1	1300	4.65	74.25	51.65	41.65	35.30	28.10	24.75	19.50	16.5	98.7
Color (Pt/Co)	3.1	566,666.66	443.08	259,237.46	101,754.75	56,105.80	27,690.25	15,512.25	13,410.0	10,387.00	9504.5	98.3

#### Biochemical oxygen demand (BOD)

BOD is one of the important parameters to check the presence of organic contaminants present in the water sample^62^. The aerobic microbes require oxygen to degrade the organic matter present in the water and BOD measures the quantity of the dissolved oxygen (DO) utilized for this decomposition. In the current research, the initial BOD value of the sludge sample and effluent was 62.2 and 1095.5 mg/L respectively. Each week (1 to 9 weeks) experimental samples were collected and analyzed. It was noticed that the BOD was decreased to 166.3, 48.37, and 25.05 mg/L on 3rd, 6th, and 9th weeks respectively. The huge decline of the BOD up to 97.7% was observed with an increase in the duration of experimental time up to 9 weeks. Table 3 and Fig. 3 illustrated changes in the BOD of simulated MB wastewater during 9 weeks of the experimental period by GBs-FBR under aerobic conditions. This reduction in the BOD was due to fixed biofilm and suspended microorganisms in the reactor^63^. It was found that the BOD removal efficiency was higher in the aerobic system^64^. Naresh et al.^26^ achieved a 98.3% reduction in BOD of MB wastewater in 9 weeks by using a TDR media biofilm reactor. Egbuikwem et al.^65^ observed approximately 87% of BOD removal under the aerobic condition in 97 days from the simulated mixture of dyes wastewater in a suspended growth reactor. In another study, the textile wastewater was treated using a biological aerated filter and it was observed that 99% of BOD removal was achieved under high organic loading rate^66^.

#### pH

The pH range of 4–9 is the most favorable for bacterial growth and its existence^67^. In this experimental work, the pH of the stock solution was adjusted to 8.45, and every week the pH of the influent and effluent from GBs-FBR were recorded regularly. A variation was observed in the pH of the sludge sample and the stock solution, i.e., the pH was at 8.18 in the 1st week and 8.28 in the 4th week and later on marginal changes between 8.30 ± 0.02 were detected. The previous research investigations illustrated that higher dye degradation takes place at neutral/basic pH values^68^. The pH was declined from 12.2 to 8 and was associated with maximum dye degradation^69^. The pH effect on the biological activity of anaerobes was investigated by conducting a batch experiment, and the pH was adjusted to 4 ± 10 by using HCL and NaOH. It was observed that after 2 h of incubation at 55 °C, the pH was found to be in the range of 6.5 ± 7.5 due to microbial activites^70^. The results of the current research work are in agreement with the previous studies and it can be concluded that the pH 8.30 has shown a good impact on simulated MB dye wastewater treatment (Table 3 and Fig. 3).

#### Ammonia (NH_3_)

NH_3_ is a colorless gas with a combination of nitrogen and hydrogen gases, and it has a high impact on various microbial communities^71^. Most of the nitrogen present in the sludge or the stock is in the form of ammonia^72^. To understand the physiological activity of the microbes, the changes in the ammonia ion concentration were studied at different week’s intervals (0–9 weeks) of the experimental operation. It was observed that the NH_3_ concentration increased with an increase in time duration (Table 3 and Fig. 3). A drastic increase in the NH_3_ concentration was observed from the obtained results till the 2nd week, from 3rd week the NH_3_ concentration has started declining, and it reached to 16.5 mg/L (98.7% decline) at the end of the operation (9th week), as shown in the Fig. 3. The initial increase of NH_3_ up till 2nd week (from 16.5 to 74.25 mg/L) might be because of the two reasons, (1) the presence of a higher concentration of NH_3_ in the stock solution and, (2) microorganisms present in the sludge sample might have produced more ammonium ions. The removal efficiency of NH_3_ depends on hydraulic loading and DO. It was found that an increase in hydraulic loading rate decreases the NH_3_ removal efficiency, while increasing the DO concentration increases the NH_3_ removal efficiency^73^. In a study on remediation of MB dye using biofilms developed on rubber media, NH_3_ concentration was reduced to 99.61% within 9 weeks^26^.

#### Color

A very high concentration of the MB dye (25 gm) was used in the preparation of a simulated MB wastewater solution. Very high variation in the color was observed in the effluent samples of GBs-FBR, collected at different time intervals during the experimental operation of 9 weeks (Table 3 and Fig. 3). It was noticed that the color intensity of the effluent sample was increased till the 2nd week (from 566,666.66 to 259,237.46 pt/Co units), and it started decreasing slowly thereafter to 9504.5 pt/Co units (98.3% reduction) by the end of 9th week. The increase in color intensity might be due to the high MB dye concentration in the stock solution. A further reduction in the color intensity might be due to the activity of the microbial community present in the sludge and also in the biofilm attached to the GBs surfaces. Similar results were reported by another researcher, where 99.8% of the color deterioration was observed in 9 weeks on treating the MB dye simulated wastewater by fixed biofilms on TDR media^26^. Moreover, the true color of simulated mixed wastewater has almost turned invisible by the suspended growth process due to the activity of microbial communities^65^. The maximum color removal can also be achieved by integrating several techniques such as ozonation, chemical coagulation, fluidized biofilm process, and electrochemical oxidation processes and also by employing the support media (like GBs, rubber, pebbles, etc.) for the biofilm development^61,74^.

### Fourier-transform infrared spectroscopic (FTIR) analysis

**Figure 4:**
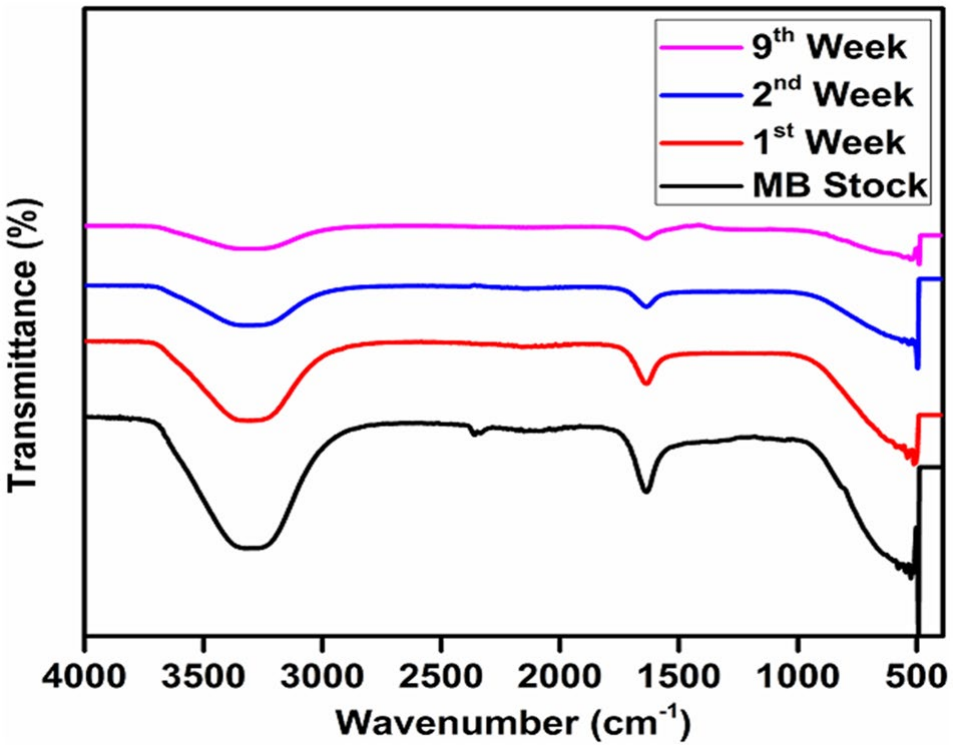
Fourier-transform infrared spectroscopic (FTIR) analysis of Methylene Blue (MB) simulated wastewater before treatment, and after 1st, 2nd and 9th-week treatment by GBs fixed biofilm reactor (FBR) under aerobic conditions.

The functional groups present in the GBs filter media, MB simulated wastewater before treatment and, after 1st, 2nd, and 9th-week treatment were analyzed by employing FTIR (Fig. 4). In the MB simulated wastewater, the absorbance peaks representing aromatic rings, identified between 1591 to 1363 cm^−1^ and ANH/AOH overlapped stretching vibration absorbance was observed at 3410 cm^−1^^75^. A fine and sharp stretching peak of MB was observed in the MB simulated wastewater and the peak went on diminishing with the time. This can be attributed to the deterioration of MB dye by various microbial communities present in the biofilms attached to the GBs filter media surface. In another research, the *Proteus mirabilis* TJ-1 was employed for the removal of hazardous Basic Blue 54 (BB54) dye from the wastewater in a bioreactor and have recorded the infrared spectra wavelengths in the range of 4000–400 cm^−1^. Further, they have identified a broad stretching intense peak around 3400 cm^−1^ which indicates the presence of amino and hydroxyl groups. Moreover, another stretching peak observed around 1700 cm^−1^, indicates carboxyl groups in the sample^76^. Thus, in light of previous research, the FTIR analysis of simulated MB wastewater has disclosed the existence of adsorbing groups like hydroxyl, carboxyl, and amino groups. Similarly, the bioflocculants obtained from biologically aerated filter backwashed sludge can be used for the treatment of dye wastewater. In another study, the FTIR spectrum of the bioflocculant was analyzed to explore the correlation between the flocculating activity and functional groups. It was found that a broad stretching peak which is a characteristic of the hydroxyl group was identified around 3371 cm^−1. ^ While, a peak at 2954 cm^−1^ is indicating a weak C–H stretching band. Two other bands around 1658 and 1415 cm^−1^ were also identified by the infrared spectra, and also reported previousely^77^.

### Characterization of glass beads (GBs) surface and biofilms by SEM–EDX

**Figure 5:**
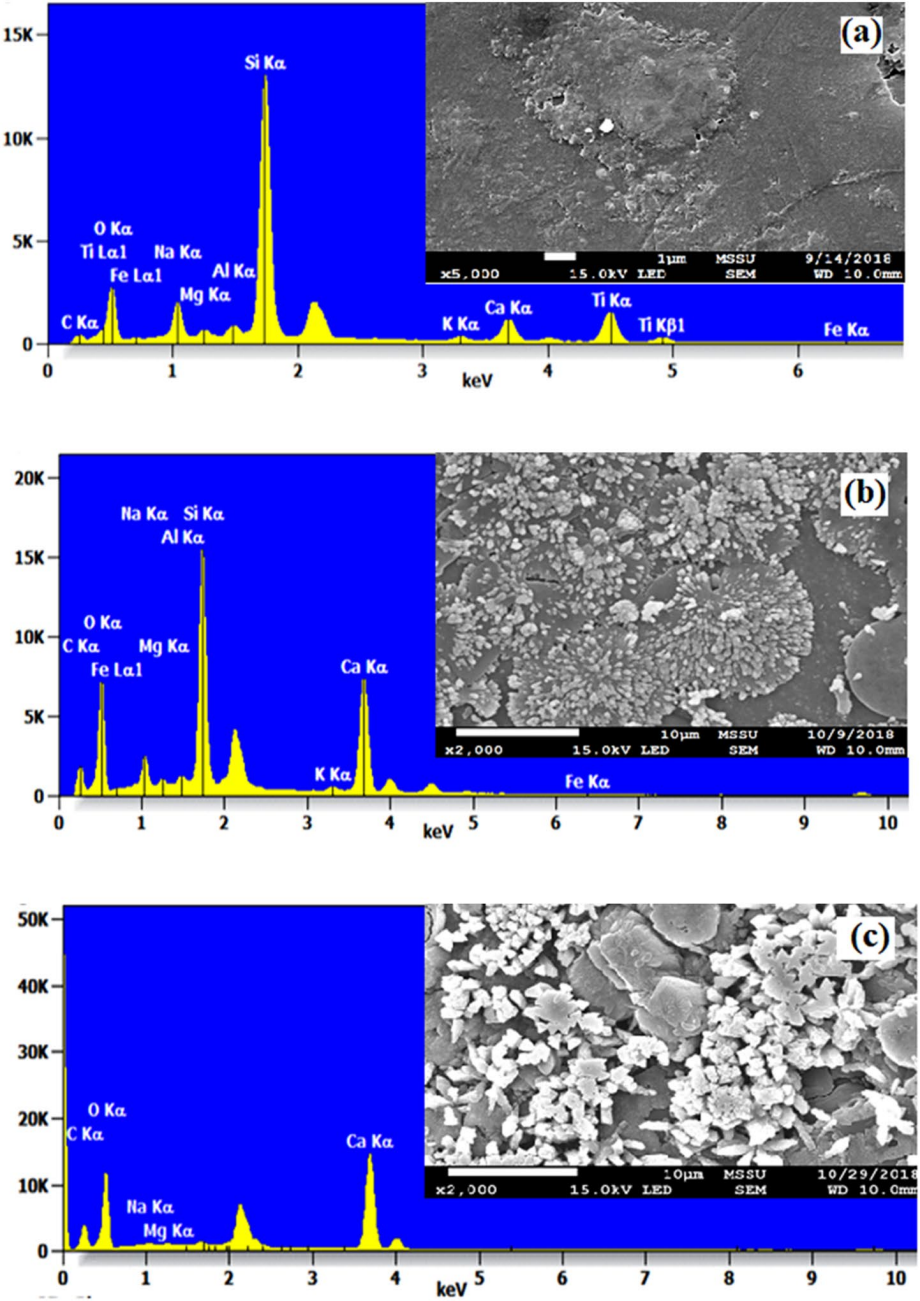
(**a**) Scanning electron micrographs (SEM) and EDX configuration of GBs without biofilm configuration (raw GB); (**b**) SEM image and EDX of GBs after 1st week of biofilm development; and (**c**) SEM image and EDX of GBs surface with 9 weeks mature aerobic biofilm.

The SEM–EDX analysis of the GBs without biofilm, and after the development of biofilm in the 1st and 9th week under aerobic conditions in the FBR during the treatment of simulated MB wastewater were shown in Fig. 5. The EDX analysis of GBs before the development of biofilms have C (3.4), O (25.6), Na (5.8), Mg (0.8), Al (1.2), Si (37.5), K (0.8), Ca (7.4), Ti (16.4), Fe (1.2) % weight respectively. It was observed that a large amount of silicon was present in the GBs filter media. The GBs filter media having 1-week-old biofilms have C (6.1), O (40.1), Na (3.6), Mg (0.4), Al (0.7), Si (22.8), K (0.5), Ca (25.2), Fe (0.6) % weight respectively. It was observed that % weight of carbon, oxygen, and calcium have increased, while % weight of the elements like Na, Mg, Al, Si, K, and Fe have decreased, as compared with the GBs without biofilms. Further, the GBs having 9 weeks old biofilms have C (5.0), O (55.1), Na (0.4), Mg (0.2), K (0.1), and Ca (39.1) % weight respectively. From the overall results, it can be concluded that the % weight of the Ca has increased and Si has decreased tremendously in 9 weeks. This might be due to the presence of calcium in the MB stock solution, which got attached to the biofilm formed on the surface of the GBs filter media. Various research investigators have revealed that the silicone is immensely susceptible to the colonization of *E. coli* and favors the formation of biofilm on the GBs surface^78–81^. In another study, pebbles, GBs, and sand were employed in a continuous flow reactor as biofilm support media for the removal of chromium (Cr (III)) from tannery effluent. It was found that 92.6% of Cr (III) was adsorbed on the biofilm that was developed on the sand biofilter and it was confirmed by the Si peak due to the interaction of bacterial consortium developed on the sand with Cr (III)^82^.

### Evaluation of degradative changes in glass beads (GBs) and in simulated Methylene Blue (MB) dye wastewater by Raman spectroscopy

**Figure 6:**
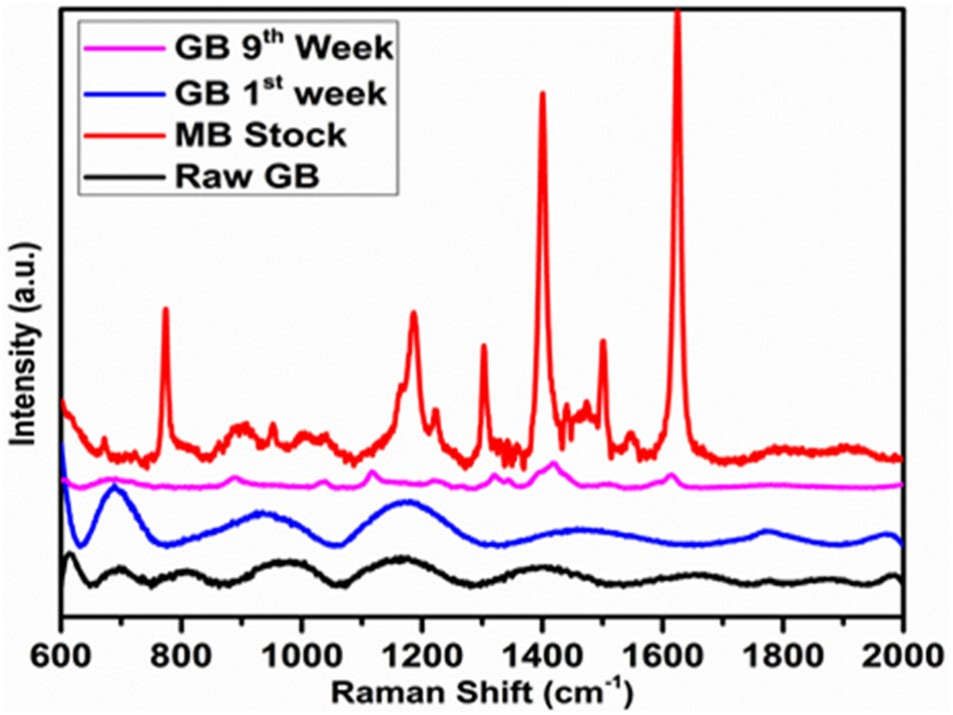
Raman spectroscopic analysis of GBs without biofilm (Raw GB), simulated MB wastewater (Raw Stock), and GB samples of 1st, and 9th-week old aerobic biofilms.

**Figure 7:**
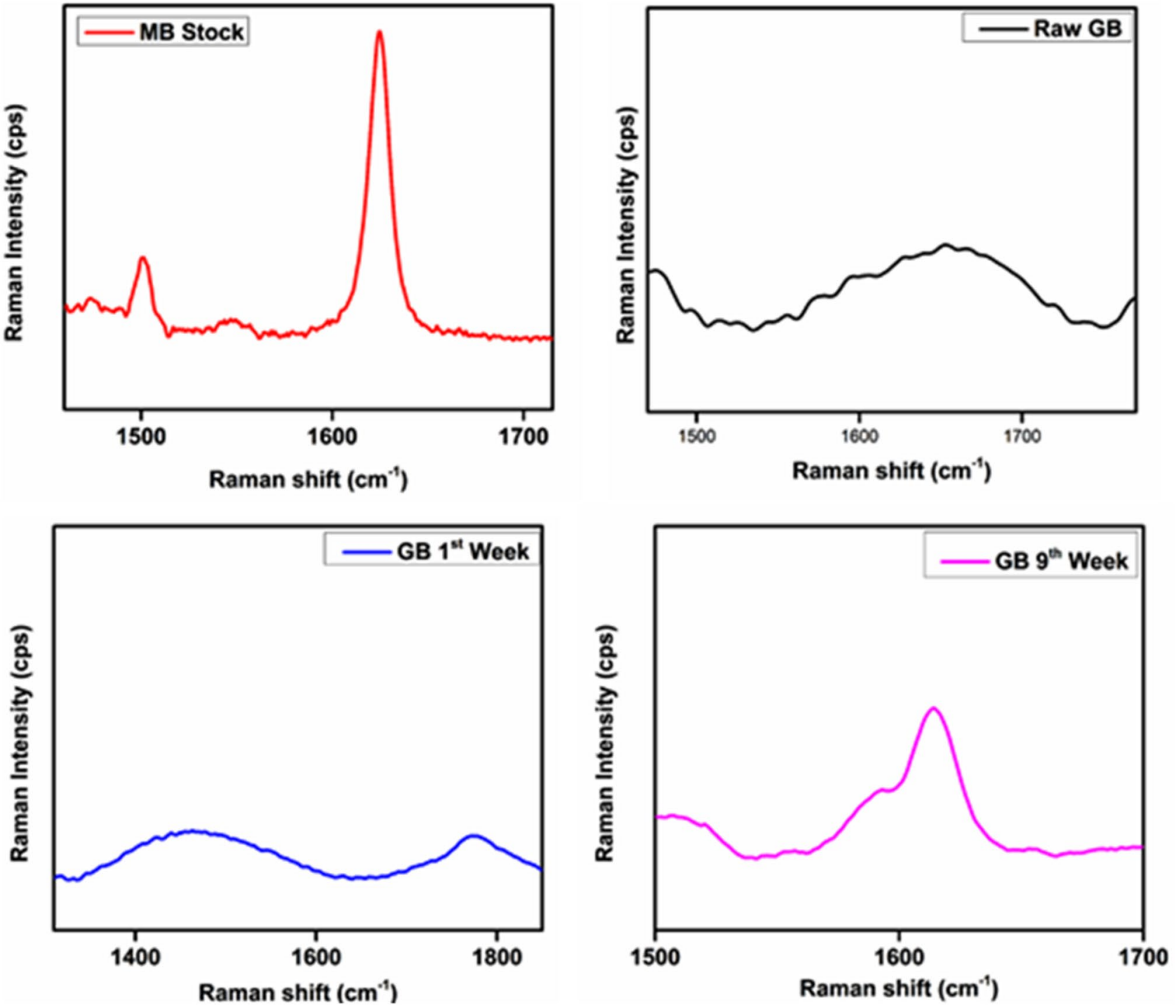
Raman spectroscopic peaks shifts between 1400 and 1700 cm^−1^ of Methylene Blue (MB) simulated wastewater, GBs without biofilm (Raw GB), and GB samples of 1st, and 9th-week old aerobic biofilms.

The chemical characterization of the biofilm matrix including extracellular polymeric substances (EPS) and microbial constituents can be characterized by using Raman microscopy^83,84^. Specific marker bands and frequency regions of various biofilm constituents can also be revealed by the examination of reference samples such as microorganisms, polysaccharides, etc.^85^. MB simulated wastewater, GBs without biofilms and, the GBs after the formation of 1st and 9th weeks' old biofilm were characterized using the Raman spectroscopy (Figs. 6, 7). From the Raman spectra data, it was clear that no bands were observed in the GBs sample without biofilms between 1500 and 1700 cm^−1^. While the characteristic Raman bands of the MB stock solution were very strong at 1618 cm^−1^. Few other peaks in the MB stock solution are indicating aromatic compounds, C = C, CH_2_, CH_3_, alicyclic and aliphatic compounds, C = S, O–O, C–S at 1600, 1500, 1400, 1300, 1190, 890, 780 Wavenumbers (cm^−1^) respectively. In the GB sample having a 1-week-old biofilm, Raman bands were week near 1800 cm^−1^ and it might be the MB band which was slightly shifted towards 1800 cm^−1^. A clear and strong Raman band of the MB dye was spotted at 1618 cm^−1^ in GBs sample having 9-week-old biofilms. In another study, Raman spectroscopy was applied for the characterization of biofilm developed by *Raoultella planticola* and *E. coli*^86^. The presence of polysaccharides and nucleic acids were revealed in the biofilm as indicated by Raman bands between the region 1,200 and 1,000 cm^−1^. Further, various structural aspects within the biofilm due to chemical amalgam can be corresponded by Raman, such as a band at 1,655 cm^−1^ observed in multispecies biofilms was appropriated to C = O (aldehyde), C = C, or C = O (keto)^86^. They have assigned peaks at 1,155 and 1,510 cm^−1^ to carotenoids and attributed peaks at 1,350 and 1,600 cm^−1^ to humic-like substances. Weak Raman bands identified at 1,128 and 1,621 cm^−1^ were appropriated to polysaccharides^86^. Moreover, Welter et al.^87^ characterized the inorganic pigments present in ancient glass beads by employing Raman spectroscopy. They observed peaks at 471, 630, and 773 cm^−1^ in sandwich glass beads which specify the presence of cassiterite and SnO_2_ as opacifier and coloring agent of the white interlayers. The bands at 1071, 1040, 957, 589, 429 cm^−1^ indicate the presence of Ca_3_(PO4)_2_. This experimental study has revealed the presence of cuprite (Cu_2_O) at 150, 186, 197, 212, 494, and 627 cm^−1^ bands respectively and this was achieved by fixing the laser beam straightly on microcrystals of the glass beads. And also the silicon–oxygen stretching bands of the SiO_4_ group were observed between 800 and 1200 cm^−1^. In one of the research studies, Protozoa was characterized by using the Surface-Enhanced Raman Scattering (SERS) which uses more discriminable peaks. At 1448 and 1336 cm^−1^ strong bands were identified and were attributed to lipids, polysaccharides, and proteins (CH_2_ and CH). Two broad bands around 1630 and 1320 cm^−1^ were observed in the aggregate sample which was attributed to humic-like substances. While peaks at 1502, 1152, and 1002 cm^−1^ represents carotenoids and also usual for colored bacteria and Raman peaks at 1650–1540 cm^−1^ were due to asymmetric carboxylate stretching vibrations^88^. From the results of the current research, it can be concluded that the MB simulated wastewater has firmly attached to the biofilm developed on the surface of the GBs filter media, as indicated by very clear, fine and a strong MB peak. Further, Raman bands at 1600, 1400, 1320, 1190, and 890 cm^−1^ correspond to various aromatic compounds such as CH_2_, CH_3_, C = S, and O–O respectively.

### Determination of degradative changes in glass beads (GBs) filter media by Attenuated total reflection (ATR)

**Figure 8:**
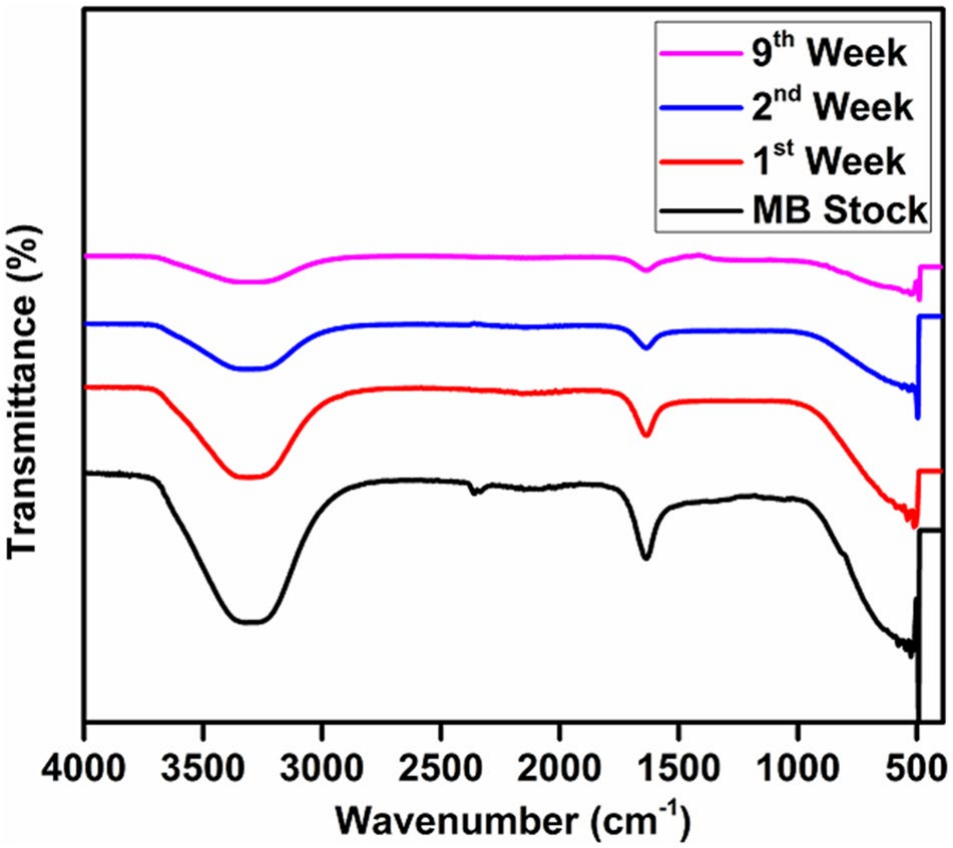
Attenuated total reflection (ATR) spectra of methylene blue (MB) stock solution, and GB samples of 1st, 2nd, and 9th-weeks' old aerobic biofilm.

ATR was used to study the complete chemical composition and the chemical reactions that take place on the surface of the media under various conditions^89^. This technique can also be utilized to estimate the metabolic activity of the microbial communities in the biofilm^90,91^. Figure 8 illustrates the ATR characterization of GBs without biofilm and GBs after the development of 1st, 2nd, and 9th weeks' old aerobic biofilm. In the raw GB media, a peak was observed at 790 cm^−1^ representing Si–C^92,93^, and the peaks 1591 cm^−1^, 1626 cm^−1^, 3290 cm^−1^ were observed in the 1st, 2nd, and 9th-week samples. The peaks observed near 1500 cm^−1^ after the development of biofilms might be due to the adsorption of MB dye in the EPS of biofilms. It was reported previously that the MB dye consists of aromatic ring structures and represented by the peak at 1591 cm^−1^ ^94^. This peak intensity has increased with an increase in the duration of treatment time till the 9th week, and it was due to the more adsorption of MB dye by biofilms. Another research work revealed that, during the primary stage of biofilm growth, EPSs were mainly generated, initiating the bacterial colonization, and have resulted in a sequential acquisition of absorption peak at 1536 cm^−1^ by ATR/FT-IR^95^. While, they also observed two more peaks at 1626 and 1536 cm^−1^, indicating Amide I and Amide II respectively, after 26 h of inoculation. Further, protein Amide I and carboxylate features were observed and confirmed in a sample collected after 118 h, which were identified in the region 1580 to 1720 cm^−1^^95^. Moreover, these visible peaks such as 1539, 1548, and 1647 cm^−1^ were also attributed by another scientific group to peptide amides and proteins (–NH_2_, –NH, C–N, C = O)^96^. Similarly, the peak 3290 cm^−1^ identified in the sample represents proteins and polysaccharides (O–H and N–H), because the stretching vibration absorbance at 3410 cm^−1^ was assigned to ANH/AOH by various researchers^25,75^.

## Conclusions

The active biofilm communities were developed on GBs filter media in the FBR system for the efficient treatment of simulated MB dye wastewater under aerobic conditions. It was observed that these metabolically active biofilms in GBs-FBR can improve the percentage removal of MB dye from the simulated MB wastewater. Various physicochemical parameters of influent like COD and BOD were reduced by 86.48 and 97.7%. The ammonia and color were reduced to 98.7 and 98.3% during experimental operation. The biofilm configuration and the variation in the elemental composition of the GBs after the development of biofilms were observed by SEM–EDX. Sharp peaks of Raman spectra indicating the presence of MB dye were observed. With an increase in the duration of operation, the MB peak intensity was decreased in the liquid samples, as confirmed by the FTIR. Similarly, ATR analysis has indicated that the MB peak intensity was increased because of the adsorption of MB dye by biofilms on the surface of GBs media. Thus, these laboratory-scale experiments of MB dye treatment using biofilms developed on GBs media have proved to be promising and therefore can be recommended for pilot trial and large-scale application. It was found that the GBs are durable and economical media, and they can be employed for the biofilm development for wastewater treatment. To improve the current appalling situation of water pollution and to protect all forms of life on this earth from synthetic dye pollution, the textile industries require effective and sustainable technologies such as the FBR systems. Additionally, recycling and reuse of water, adopting eco-friendly technologies, organizing peoples' awareness campaigns to buy environment-friendly textiles, bringing and implementing some stringent environmental laws are some of the best interventions to curb the textile industrial pollution in many countries around the world.

## Abbreviations

GBs: Glass beads
MB: Methylene blue
COD: Chemical oxygen demand
BOD: Biological oxygen demand
XPS: X-ray photoelectron spectroscopy
SEM: Scanning electron microscope
EDX: Energy dispersive X-ray spectroscopy
FBR: Fixed biofilm reactor
EPS: Extracellular polymeric substance
FTIR: Fourier transform infrared
ATR: Attenuated total reflection
